# Rapid, Simple and Cost-Effective Molecular Method to Differentiate the Temperature Sensitive (ts^+^) MS-H Vaccine Strain and Wild-Type *Mycoplasma synoviae* Isolates

**DOI:** 10.1371/journal.pone.0133554

**Published:** 2015-07-24

**Authors:** Zsuzsa Kreizinger, Kinga Mária Sulyok, Alexandra Pásztor, Károly Erdélyi, Orsolya Felde, János Povazsán, László Kőrösi, Miklós Gyuranecz

**Affiliations:** 1 Institute for Veterinary Medical Research, Centre for Agricultural Research, Hungarian Academy of Sciences, Budapest, Pest, Hungary; 2 Veterinary Diagnostic Directorate, National Food Chain Safety Office, Budapest, Hungary; 3 Rhone-Vet Kft., Herceghalom, Pest, Hungary; Universidad Nacional de La Plata, ARGENTINA

## Abstract

*Mycoplasma synoviae* infection in chickens and turkeys can cause respiratory disease, infectious synovitis and eggshell apex abnormality; thus it is an economically important pathogen. Control of *M*. *synoviae* infection comprises eradication, medication or vaccination. The differentiation of the temperature sensitive (ts^+^) MS-H vaccine strain from field isolates is crucial during vaccination programs. Melt-curve and agarose gel based mismatch amplification mutation assays (MAMA) are provided in the present study to distinguish between the ts^+^ MS-H vaccine strain, its non-temperature sensitive re-isolates and wild-type *M*. *synoviae* isolates based on the single nucleotide polymorphisms at nt367 and nt629 of the *obg* gene. The two melt-MAMAs and the two agarose-MAMAs clearly distinguish the ts^+^ MS-H vaccine strain genotype from its non-temperature sensitive re-isolate genotype and wild-type *M*. *synoviae* isolate genotype, and no cross-reactions with other *Mycoplasma* species infecting birds occur. The sensitivity of the melt-MAMAs and agarose-MAMAs was 10^3^ and 10^4^ copy numbers, respectively. The assays can be performed directly on clinical samples and they can be run simultaneously at the same annealing temperature. The assays can be performed in laboratories with limited facilities, using basic real-time PCR machine or conventional thermocycler coupled with agarose gel electrophoresis. The advantages of the described assays compared with previously used methods are simplicity, sufficient sensitivity, time and cost effectiveness and specificity.

## Introduction

*Mycoplasma synoviae* can cause respiratory disease, infectious synovitis and eggshell apex abnormality in chickens and turkeys, thus the bacterium has economic importance in poultry industry. *M*. *synoviae* infection may occur from sub-clinical to severe forms, and the clinical signs change markedly when *Mycoplasma* infection is associated with other pathogens [1–3]. *M*. synoviae has a worldwide distribution and its occurrence is increasing. Less attention paid to control programs, biosecurity lapses at farms and large concentration of poultry in small geographic areas could accelerate the spread of *M*. *synoviae* infection [4]. *M*. *synoviae* may be transmitted either vertically through the eggs, or laterally by direct contact or indirect contact via the environment [1,5]. There are three general aspects in the control of *M*. *synoviae* infection: eradication and maintaining pathogen-free status by prevention, medication or vaccination [4].

In situations where maintaining flocks free of *M*. *synoviae* is not feasible (e.g. at a multi-age commercial layer farm) vaccination is a practical option for infection control. The commercially available live attenuated vaccine contains the temperature sensitive (ts^+^) MS-H strain (Vaxsafe MS-H, Bioproperties Pty. Ltd. Australia), and it is used in many countries [6,7]. To perform control and eradication programs, molecular typing techniques have to be able to differentiate the ts^+^ MS-H vaccine strain from field isolates. It is also important to examine whether the vaccine strain has successfully colonized the respiratory mucosa and thus produced an efficient immune response against wild-type strains. The sequence analysis of the *vlhA* gene was widely used to differentiate the MS-H vaccine strain from clinical isolates [8–11]. Unfortunately, it turned out that the *vlhA* gene sequence profile of the MS-H vaccine strain is not unique and several Australian and European field strains share the same *vlhA* gene sequence [9,12].

Shahid and co-workers [13] discovered two single nucleotide polymorphisms (SNP) on the *obg* gene sequence, namely the A-G substitution at nt367 and C-T substitution at nt629 which are able to differentiate the ts^+^ MS-H vaccine strain, its rare non-temperature sensitive (ts^-^) MS-H re-isolates and wild-type *M*. *synoviae* isolates. They developed four PCRs followed by high-resolution melting (HRM)-curve analysis for the differentiation of strains based on these SNPs [7]. Although these PCR-HRM assays work well, we think they can be simplified and new assays can be designed for efficient use on basic laboratory equipment. Therefore in our study we provide melt-curve and agarose gel based mismatch amplification mutation assays (MAMA) to distinguish the ts^+^ MS-H vaccine strain, ts^-^ MS-H re-isolates and wild-type *M*. *synoviae* isolates based on the substitutions at nt367 and nt629 of the *obg* gene.

## Methods

MAMAs are used for SNP typing in many different pathogens. The detailed description of the method is presented elsewhere [14]. In brief, MAMAs are based on allele-specific primers that are SNP specific at the 3’end. A single base mismatch at the ante-penultimate (-3) position of each allele-specific primer enhances the SNP discrimination capacity of these assays. One allele-specific primer possesses an additional 15-20bp GC-clamp at the 5’end with a sequence of CGGGG and the other allele primer has no additional sequence. The GC-clamp increases the melting temperature (T_m_) of the resulting PCR product by 3–5°C, a shift that is detectable by fluorescent dye on a real-time PCR platform (Melt-MAMA) and it increases the size of the PCR product, resulting in a size difference which can also be detected by 3% agarose gel electrophoresis (Agarose-MAMA).

In the present study MAMAs were designed and tested to detect the nt367 and nt629 SNPs in the *obg* gene of *M*. *synoviae*. The genome locations, primer sequences, annealing and melting temperatures for these assays can be found in Tables 1 and 2. Melt-MAMA PCR reactions were performed in 10 μl total volume, containing 1μl target DNA diluted in 2 μl 5X Color-less GoTaq Flexi Buffer (Promega Inc., Madison, WI), 1 μl MgCl_2_ (25mM), 0.3 μl dNTP (10 mM, Qiagen Inc., Valencia, CA), 0.5 μl EvaGreen (Biotium Inc., Hayward, CA), primers (10 pmol/μl) according to Table 1 and 0.08 μl GoTaq DNA polymerase (5 U/μl; Promega). Melt-MAMAs were performed on an Applied Biosystems Step-One Plus real-time PCR system with StepOne Softwarev2.2.2. Thermocycling parameters were 95°C for 10 min, followed by 39 cycles of 95°C for 15 sec and 58°C for 1 min. Endpoint PCR products were subjected to melt analysis using a dissociation protocol comprising 95°C for 15 sec, followed by incremental temperature ramping (0.2°C) from 58°C to 95°C. EvaGreen fluorescent intensity was measured at 525 nm at each ramp interval and plotted against temperature.

**Table 1:** SNP locations in the *obg* gene, SNP state, primer sequences, primer volumes and thermal cycler parameters for the two MS-H melt-MAMAs.

Assay name	SNP position	SNP state	MAMA primer names	MAMA primer sequences	Primer (10 pmol/µl) volume in 10 µl reaction mixture (µl)	Annealing temperature (°C)	Melting temperature (°C)
MS-H1	367	G/A	MS-H1-poz	ggggcggggcggggcGCTAAAGGCGGAAAAGtCa	0.600	58	80.1
			MS-H1-neg	GCTAAAGGCGGAAAAGaCg	0.150		75.0
			MS-H1-R	GGCAATTCTAGGAGCGGT	0.150		
MS-H2	629	C/T	MS-H2-poz	ggggcggggcggggCTTTAATAAGYCCAGGAAGATaCg	0.150	58	76.8
			MS-H2-neg	CTTTAATAAGYCCAGGAAGATtCa	0.150		70.9
			MS-H2-R	CCTTAGTTCCTCAGTTAGGTCTTG	0.150		

**Table 2:** SNP locations in the *obg* gene, SNP state, primer sequences and primer volumes for the two MS-H agarose-MAMAs.

Assay name	SNP position	SNP state	MAMA primer names	MAMA primer sequences	Primer (10 pmol/µl) volume in 25 µl reaction mixture (µl)	Annealing temperature (°C)
MS-H1	367	G/A	MS-H1-poz-agarose	ggggcggggcggggcggggcGCTAAAGGCGGAAAAGtCa	3	58
			MS-H1-neg	GCTAAAGGCGGAAAAGaCg	1	
			MS-H1-R	GGCAATTCTAGGAGCGGT	1	
MS-H2	629	C/T	MS-H2-poz-agarose	ggggcggggcggggcggggCTTTAATAAGYCCAGGAAGATaCg	1	58
			MS-H2-neg	CTTTAATAAGYCCAGGAAGATtCa	3	
			MS-H2-R	CCTTAGTTCCTCAGTTAGGTCTTG	1	

Agarose-MAMAs were performed in Biometra–T Personal thermal cycler (Biometra Inc., Göttingen, Germany) in 25 μl total volume, containing 1 μl target DNA diluted in 5 μl 5X Green GoTaq Flexi Buffer (Promega Inc., Madison, WI), 2.5 μl MgCl_2_ (25mM), 0.5 μl dNTP (10 mM, Qiagen Inc., Valencia, CA), primers (10 pmol/μl) according to Table 2 and 0.25 μl GoTaq DNA polymerase (5 U/μl; Promega). Thermocycling parameters were 94°C for 5 min, followed by 40 cycles at 94°C for 30 sec, 58°C for 30 sec and 72°C for 30 sec. The final elongation step was performed at 72°C for 5 min. Electrophoresis was performed in 3% agarose gel (MetaPhor Agarose, Lonza Group Ltd., Basel, Switzerland) and a 20-bp DNA ladder (O'RangeRuler 20 bp, Thermo Fisher Scientific Inc.) was used as molecular weight marker.

**Fig 1:**
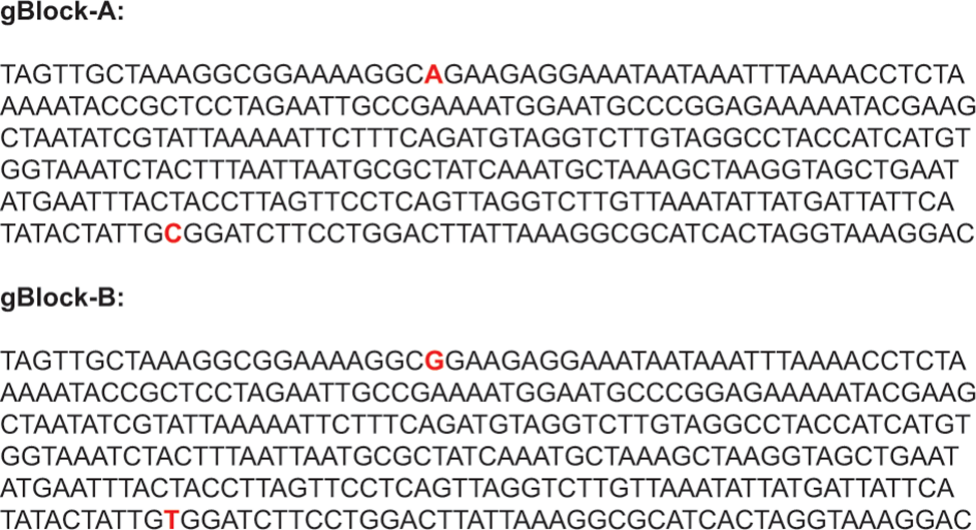
Sequences of the gBlocks used as positive and validation controls in the study. The gBlocks (Integrated DNA Technologies Inc., Coralville, IA) contain 330 bp long fragment of the *obg* gene between nt342 and nt672.

In order to test the sensitivity of the assays, 10 fold dilutions of gBlocks (Integrated DNA Technologies Inc., Coralville, IA) containing 200 ng of a 330 bp long fragment of the *obg* gene (from nt342 to nt672) were used (Fig 1). The specificity of the assays was tested by including the following avian *Mycoplasma* species in the analysis: *M*. *anatis*, *M*. *anseris*, *M*. *columbinasale*, *M*. *columborale*, *M*. *cloacale*, *M*. *gallinaceum*, *M*. *gallinarum*, *M*. *gallisepticum*, *M*. *gallopavonis*, *M*. *iners*, *M*. *iowae*, *M*. *meleagridis* and *M*. sp. 1220. The temperature sensitive (ts^+^) MS-H vaccine strain (Vaxsafe MS-H), *M*. *synoviae* type strain (WVU 1835, NCTC 10124), seven clinical *M*. *synoviae* isolates (3 chicken and 4 turkey isolates), nine ts^+^ MS-H re-isolates and trachea swabs taken from 40 vaccinated and 40 clinically infected (seropositive) chickens were included in the analysis as well (Table 3). DNA was extracted from the isolated strains and trachea swabs with the Qiamp DNA Mini kit (Qiagen GmbH, Hilden, Germany). Ethical approval was not required for the study as all samples and strains were collected by the authors during routine diagnostic examinations.

**Table 3:** Background information of the *Mycoplasma synoviae* strains, ts^+^ MS-H re-isolates and trachea swab samples included in this study.

Sample ID	Sample type	Sample source	Location of sampled farm in Hungary	Collection date
MS 1	*Mycoplasma synoviae* strain	chicken trachea	Széchényfelfalud	2014
MS 2	*Mycoplasma synoviae* strain	chicken trachea	Szentmártonkáta	2015
MS 3	*Mycoplasma synoviae* strain	chicken trachea	Jászapáti	2015
MS 4	*Mycoplasma synoviae* strain	turkey trachea	Völcsej	2014
MS 5	*Mycoplasma synoviae* strain	turkey trachea	Simaság	2014
MS 6	*Mycoplasma synoviae* strain	turkey trachea	Ács	2014
MS 7	*Mycoplasma synoviae* strain	turkey trachea	Bőny	2014
MS-VR1-VR5	ts^+^ MS-H re-isolates	chicken trachea	Tárkány (farm-1-5)	2015
MS-VR6-VR9	ts^+^ MS-H re-isolates	chicken trachea	Bábolna (farm-1-4)	2015
MS S1-S10	trachea swabs from seropositive animals	chicken trachea	Széchényfelfalud	2014
MS S11-S20	trachea swabs from seropositive animals	chicken trachea	Szentmártonkáta	2015
MS S21-S30	trachea swabs from seropositive animals	chicken trachea	Jászapáti	2015
MS S31-S40	trachea swabs from seropositive animals	chicken trachea	Hegyeshalom	2015
MS V1-V10	trachea swabs from vaccinated animals	chicken trachea	Tárkány (farm-1)	2015
MS V11-V20	trachea swabs from vaccinated animals	chicken trachea	Tárkány (farm-2)	2015
MS V21-V30	trachea swabs from vaccinated animals	chicken trachea	Bábolna (farm-1)	2015
MS V31-V40	trachea swabs from vaccinated animals	chicken trachea	Bábolna (farm-2)	2015

## Results

**Fig 2:**
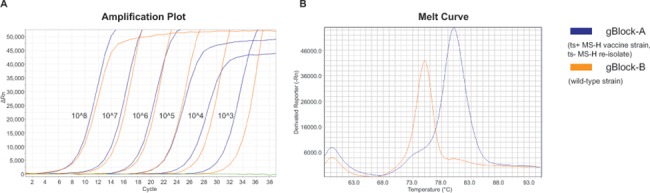
Amplification plot and melting-curves of MS-H1 melt-MAMA. Amplification plot (**A**) of dilution series of gBlock validation controls showing the sensitivity of the MS-H1 assay. Green line represents negative control. Melting curves (**B**) show melting temperatures for the temperature sensitive MS-H vaccine strain and non-temperature sensitive MS-H re-isolate (Tm: 80.1°C; blue line) or wild-type strain (Tm: 75.0°C; orange line).

**Fig 3:**
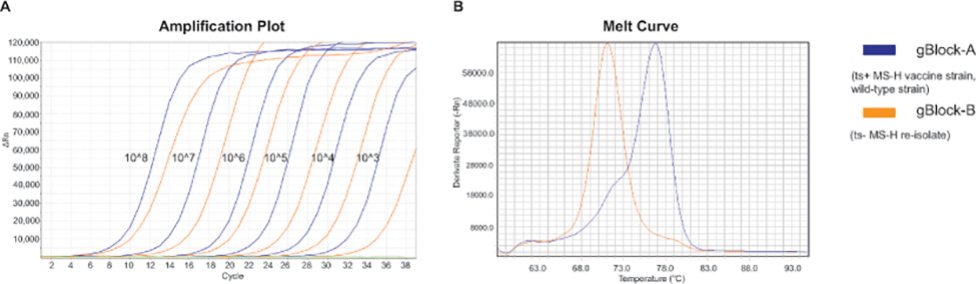
Amplification plot and melting-curves of MS-H2 melt-MAMA. Amplification plot (**A**) of dilution series of gBlock validation controls showing the sensitivity of the MS-H2 assay. Green line represents negative control. Melting curves (**B**) show melting temperatures for the temperature sensitive MS-H vaccine strain and a wild-type strain (Tm: 76.8°C; blue line) or non-temperature sensitive MS-H re-isolate (Tm: 70.9°C; orange line).

**Fig 4:**
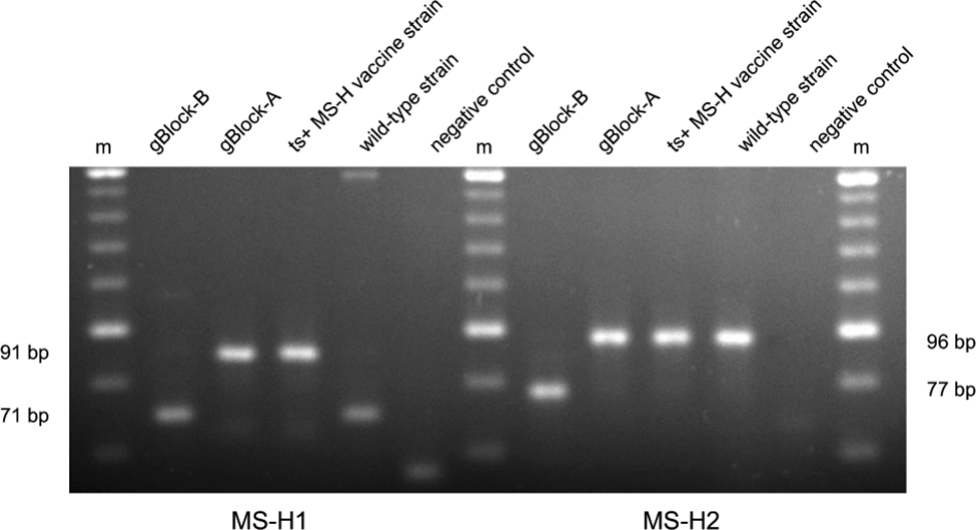
PCR product sizes of MS-H1 and MS-H2 agarose-MAMAs in agarose gel. Electrophoresis was performed in 3% agarose gel (MetaPhor Agarose, Lonza Group Ltd., Basel, Switzerland) and a 20-bp DNA ladder (O'RangeRuler 20 bp, Thermo Fisher Scientific Inc.) was used as molecular weight marker (m). In the MS-H1 assay gBlock-B and a wild-type strain yielded 71 bp fragments, while gBlock-A and the temperature sensitive MS-H vaccine strain produced 91 bp fragments. In the MS-H2 assay gBlock-B (non-temperature sensitive MS-H re-isolate homologue) yielded 77 bp fragment, while gBlock-A, the temperature sensitive MS-H vaccine strain and wild-type strains give 96 bp fragments.

Both melt-MAMAs clearly differentiated the ts^+^ MS-H vaccine strain genotype, ts^-^ MS-H re-isolate genotype and wild-type *M*. *synoviae* strain genotype (Table 4). The MS-H1 assay (nt367 SNP position) distinguished ts^+^ MS-H vaccine strain and ts^-^ MS-H re-isolates from wild-type *M*. *synoviae* isolates with 80.1°C and 75.0°C melting temperatures, respectively (Fig 2). The MS-H2 assay (nt629 SNP position) distinguished ts^+^ MS-H vaccine strain and wild-type *M*. *synoviae* strains from ts^-^ MS-H re-isolates with 76.8°C and 70.9°C melting temperatures, respectively (Fig 3). The agarose-MAMA versions of the MS-H1 and MS-H2 assays also differentiated the ts^+^ MS-H vaccine strain genotype, ts^-^ MS-H re-isolate genotype and wild-type *M*. *synoviae* strain genotype based on the 19-20bp fragment size differences of the PCR products obtained from certain genotypes (Table 4 and Fig 4).

**Table 4:** Matrix showing the SNP states, melt-MAMA melting temperatures (T_m_) and agarose-MAMA PCR fragment sizes in the ts^+^ MS-H vaccine strain genotype, ts^-^ MS-H re-isolate genotype and wild-type *M*. *synoviae* strain genotype.

Strain genotypes	nt367 SNP state	nt629 SNP state	MS-H1 melt-MAMA Tm (°C)	MS-H2 melt-MAMA Tm (°C)	MS-H1 agarose-MAMA PCR fragment size (bp)	MS-H2 agarose-MAMA PCR fragment size (bp)
ts^+^ MS-H vaccine strain	A	C	80.1	76.8	91	96
ts^-^ MS-H re-isolate^a^	A	T	80.1	70.9	91	77
wild-type strain^b^	G	C	75.0	76.8	71	96

^a^and rare 94036-2-1a genotype ts^+^ MS-H re-isolates [7,13].

^b^and MS-H^4^ like genotype ts^-^ MS-H re-isolates [7,13].

The sensitivity of both MS-H1 and MS-H2 melt-MAMAs was 10^3^ copy numbers, while both the MS-H1 and MS-H2 agarose-MAMAs showed a sensitivity of 10^4^ copy numbers. Neither assay cross-reacted with the other tested avian *Mycoplasma* species. The *M*. *synoviae* type strain (WVU 1835, NCTC 10124), the seven clinical *M*. *synoviae* isolates and 35 out of 40 trachea swabs taken from clinically infected chickens showed the wild-type strain profile while the ts^+^ MS-H vaccine strain (Vaxsafe MS-H), the nine ts^+^ MS-H re-isolates and 38 out of 40 trachea swabs taken from vaccinated chickens showed a ts^+^ MS-H vaccine strain profile in the melt and agarose-MAMAs. These results were confirmed by sequencing of the *obg* gene of these samples as well (data not shown).

## Discussion

*M*. *synoviae* causes infectious synovitis, airsacculitis and eggshell apex abnormality in chickens and turkeys, which eventually result in significant economic losses. The ts^+^ MS-H vaccine strain is used worldwide in order to reduce economic losses and to eliminate wild-type *M*. *synoviae* strains from poultry farms. The ability to differentiate the vaccine strain from wild-type isolates is essential for the evaluation of the effect of vaccination and eradication programs.

Micro-titrating, incubating and quantifying the isolated strains at two different temperatures in order to differentiate the ts^+^ MS-H vaccine strain from the ts^-^ MS-H re-isolates and wild-type strains is time-consuming [6,15]. The sequence analysis of the *vlhA* gene with different assays is a widely used method to differentiate *M*. *synoviae* isolates. However, this is also a relatively time-consuming and expensive method, but its biggest pitfall is that it is unable to distinguish several Australian and European wild-type *M*. *synoviae* isolates and ts^-^ MS-H re-isolates from the ts^+^ MS-H vaccine strain [7,9,12].

Shahid and co-workers [7,13] discovered two specific SNPs on the *obg* gene and developed HRM-curve analysis based assays to differentiate the ts^+^ MS-H vaccine strain, ts^-^ MS-H re-isolates and wild-type *M*. *synoviae* strains. In the current study we presented novel genotyping assays targeting these two SNPs on the *obg* gene which clearly differentiate the ts^+^ MS-H vaccine strain, ts^-^ MS-H re-isolates and wild-type *M*. *synoviae* strains. Our assays are specific and sensitive enough (10^3^−10^4^ copy numbers) to detect and describe *M*. *synoviae* strains, when wild or vaccine strains are present as single specific pathogen in the sample. We also think that our assays are preferable to the assays developed earlier for the following three reasons: they are rapid, they can be performed directly on clinical samples (e.g. swabs) and the assays can be run simultaneously at the same annealing temperature. They are also simple as they can be performed on basic real-time PCR machines (without the HRM-curve analysis function) and on conventional PCR equipment coupled with agarose gel electrophoresis. They are cost effective as they do not require the expensive culture process, PCR product sequencing or costly reagents (e.g. TaqMan probes).

Unfortunately, besides the above listed advantages, the presented methods also have their pitfalls similarly to previous assays based on the *obg* gene. Namely, the MS-H^4^ like genotype ts^-^ MS-H re-isolates show the same *obg* gene profile as wild-type isolates, and the rare 94036-2-1a genotype ts^+^ MS-H re-isolates show the same *obg* gene profile as the average ts^-^ MS-H re-isolates. These re-isolates can only be ascertained by using *vlhA* gene based HRM assay in combination with *obg* gene based assays (Table 4) [7,9,13]. Another pitfall is that the genotyping of mixed infections (e.g. wild type strain superinfected vaccine strain) is unreliable because of the characteristics of the MAMA method (e.g. competing primers).

We hope that the presented assays will be helpful for both well-equipped laboratories and those with basic facilities throughout the world to differentiate the ts^+^ MS-H vaccine strain, ts^-^MS-H re-isolates and wild-type *M*. *synoviae* strains and thus facilitate *M*. *synoviae* control programs.
